# Effect of a Prolonged-Release System of Urea on Nitrogen Losses and Microbial Population Changes in Two Types of Agricultural Soil

**DOI:** 10.1021/acsomega.3c04572

**Published:** 2023-10-31

**Authors:** Carlos Gregorio Barreras-Urbina, Francisco Rodríguez-Félix, José Luis Cárdenas-López, Maribel Plascencia-Jatomea, Manuel Pérez-Tello, Ana Irene Ledesma-Osuna, Tomás Jesús Madera-Santana, José Agustín Tapia-Hernández, Daniela Denisse Castro-Enríquez

**Affiliations:** † Departamento de Investigación y Posgrado en Alimentos (DIPA), Universidad de Sonora, Hermosillo, Sonora 83000 Mexico; ‡ Departamento de Ingeniería Química y Metalurgia, Universidad de Sonora, Hermosillo, Sonora 83000 Mexico; § Centro de Investigación en Alimentación y Desarrollo, A. C., Coordinación de Tecnología de Alimentos de Origen Vegetal, Carretera Gustavo Enrique Astiazarán Rosas Núm. 46. La Victoria, C.P., 83304 Hermosillo, Sonora México

## Abstract

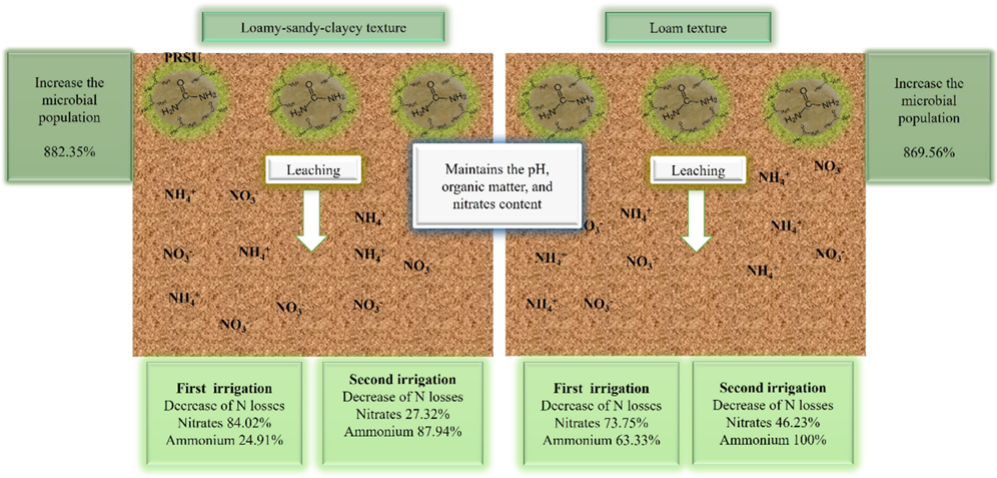

Urea is the nitrogen-containing fertilizer most used in agricultural fields; however, the nutrient given by the urea is lost into the environment. The aim of this research was to determine the effect of two soil textures by applying a prolonged-release system of urea (PRSU) on the N losses. This research shows an important decrease of the nitrate and ammonium losses from 24.91 to 87.94%. Also, the microbiological population increases after the application of the PRSU. It was concluded that both soil textures presented the same loss-reduction pattern, where the N from the nitrates and ammonium was reduced in the leachates, increasing the quality of the soil and the microbial population in both soil textures after the PRSU application.

## 1 Introduction

Nitrogen is an essential nutrient for food production in agricultural fields because it is an important factor for suitable plant growth.^1^ Urea is one of the nitrogen-based fertilizers that is most widely used. Its use is estimated at 70 million metric tons per year and may be doubled by the year 2050.^2^ Current fertilizer application practices show limitations; as a result, the nitrogen is not utilized by the plant, and this leads to a significant loss of nutrients to the environment.^3^ The efficiency of nitrogen fertilizers such as urea ranges from 30 to 40%, which indicates that the rest is lost into the environment by biogeochemical processes such as ammonia (NH_3_) volatilization, nitrate (NO_3_^–^) leaching, and the loss of nitrous oxide (N_2_O) into the atmosphere.^4,5^

Urea is transformed to ammonium carbonate ((NH_4_)_2_CO_3_) in the presence of the urease enzyme and water. The ammonium carbonate undergoes an ammonification process to obtain ammonium (NH_4_^+^).^6,7^ Then, the nitrification process is carried out, which is a reduction of ammonium, in which ammonium oxidizing bacteria associated with the ammonium monooxygenase enzyme for the production of nitrites (NO_2_^–^) are involved.^6,7^ The transformation of nitrites into nitrates (NO_3_^–^) is catalyzed by the bacterium belonging to the genus *Nitrobacter*.^6^ Considering that nitrate is a suitable nitrogen form for plants, this process is beneficial for plant growth.

However, at low oxygen concentrations and in the presence of facultative anaerobic microorganisms, nitrates are transformed into molecular nitrogen and nitrous oxide that are lost into the atmosphere by volatilization.^7^ In turn, the ammonium produced enters the nitrification process, and a certain part of it, under basic pH conditions of the agricultural soil and in the presence of the enzyme urease, is converted into ammonia, which is easily volatilized. Recent studies have found that anaerobic ammonium oxidation (anammox) through autotrophic anammox bacteria can oxidize the ammonia directly into molecular nitrogen without the release of nitrous oxide into the environment.^8^

During the processes described previously, nitrogen forms are easily lost into the environment, including NO_3_^–^ and NH_4_^+^, which are absorbed by the plant for suitable nutrition, whereby the loss of nitrogen leads to an inefficiency in its use, reduction of biomass, environmental pollution, decrease in the yields, low-quality products, and consequently, economic problems for farmers.^3,9^

Currently, the use of prolonged-release systems has been studied as an alternative to avoid environmental damage, decrease economic losses, and increase quality products.^10^ For agricultural applications of these systems, they are required to be biodegradable and eco-friendly. Wheat gluten is one of the most important natural polymers in the agri-food industry. It is economical, biodegradable, and biocompatible, as well as being easily obtainable as a byproduct from the isolation of starch.^11,12^

Castro-Enríquez et al.^13^ developed wheat gluten membranes loaded with urea and determined the effectiveness of gluten as a matrix for retention and subsequent urea release. Barreras-Urbina et al.^10^ developed microspheres loaded with urea by the nanoprecipitation process. The authors showed the potential application of this wheat gluten system for the release of urea into agricultural fields. Castro-Enríquez et al.^14^ developed microparticles of wheat glutenins by the electrospray method. The authors conducted the physicochemical characterization and found suitable results to suggest the potential application of glutenin microparticles as a controlled-release system of urea. In recent years, Gao et al.^15^ used a coated urea polymer and coated urea sulfur, and the authors measured the effectiveness of the release system in agricultural soils.

While several investigations have been conducted, there are no reports on a similar system to reduce nitrogen losses into the environment. Current studies have been focused on the development of materials with a barrier function to decrease water contact, which leads to prolonged release. Li et al.^16^ tested three types of controlled-release systems of urea (CRU) in order to test their effectiveness. The authors explain that several materials for the development of these systems have been employed such as polyurethane and water-based polymer coatings. However, the authors utilized three types of CRU and concluded that CRU reduce nitrogen losses through NH_3_ volatilization, these CRU maintaining the rice yields. Li et al.^17^ evaluated the application of polymeric coating urea (PCU). The authors found that by using a coating of urea in comparison with the conventional urea treatment, it is possible to decrease NH_4_–N in water and also maintain and increase the rice yields and N uptake by the plants. However, it has been reported that several coated materials, such as slow-release fertilizers and controlled-release fertilizers, present low availability of nitrogen; in addition, these systems are affected by temperature and microbial activities.^18^

Currently, the development of these systems is important to avoid environmental issues, due to the fact that N losses from agricultural soils comprise one of the main pollutants from agricultural practices worldwide, which gives rise to issues such as water and soil eutrophication; these losses are related with the “greenhouse” effect.^19−21^ The aim of this research was to study the effectiveness of PRSU to reduce N losses, the effect on two agricultural soil textures located in the State of Sonora, Mexico, and to discuss its relation to the microbial population and PRSU biodegradability under environmental conditions.

## 2 Materials and Methods

### 2.1 Preparation of the Prolonged-Release System of Urea (PRSU)

The PRSU was prepared using 0.55 g of commercial wheat gluten (Rockette) mixed with urea (Fagalab) solution 1M; the samples were frozen and then lyophilized. The specific methodology of the preparation and characterization of the material has been previously published.^22^

### 2.2 Sampling and the Physicochemical Properties of Agricultural Soil

The experiments were carried out using two types of agricultural soils from different locations in the state of Sonora, Mexico: (1)Valley of Empalme (VE) (28°08′21.0″N 110°41′43.9″W) with soil texture of loam and (2) experimental fields in the Agricultural and Livestock Department of the University of Sonora (ALDUS) (Km 21 toward the Coast of Hermosillo (29°00′52.5″N 111°08′03.9″W)) with soil texture of loamy-sandy-clayey. The soils from both locations were sampled by using a diagonal sampling method according to the Official Mexican Standard (NOM-021-SEMARNAT-2000). Twenty-five samples were collected for each soil location from a depth ranging from 25 to 30 cm. After this, the samples were thoroughly mixed to form a single sample for each site, and their physical and chemical properties were determined by sending them to a local specialized laboratory; the methodologies were based on the DTPA (diethylenetriamine triamine pentaacetic acid) and EDTA (ethylenediaminetetraacetic acid) acid methods, and for S, it was done using KCL. The methodologies were based on the DTPA and EDTA acid methods, which are methods used to determine metals through the formation of colorimetric complexes. Table 1 presents the physicochemical properties of the soils before the study, i.e., the first measurement.

**Table 1:** Initial Physicochemical Characteristics of Loam and Loamy-Sandy-Clayey Soil Textures in the Study

parameter	loam texture	loamy-sandy-clayey texture
pH	7.06	7.29
organic matter (%)	0.54	0.67
clay (%)	29.00	23.00
silt (%)	26.00	32.00
sand (%)	45.00	45.00
NO_3_^–^ (ppm)	155.00	258.54
K (ppm)	105.00	96.00
Ca (ppm)	2,690.00	2,950.00
Mg (ppm)	280.00	220.00
S (ppm)	20.00	17.00
Fe (ppm)	1.80	1.70
Cu (ppm)	1.60	1.40
Zn (ppm)	2.20	1.30
Mn (ppm)	1.20	1.10
Na (ppm)	570.00	189.00

### 2.3 Leaching Experiment and Nitrogen Losses

**Figure 1:**
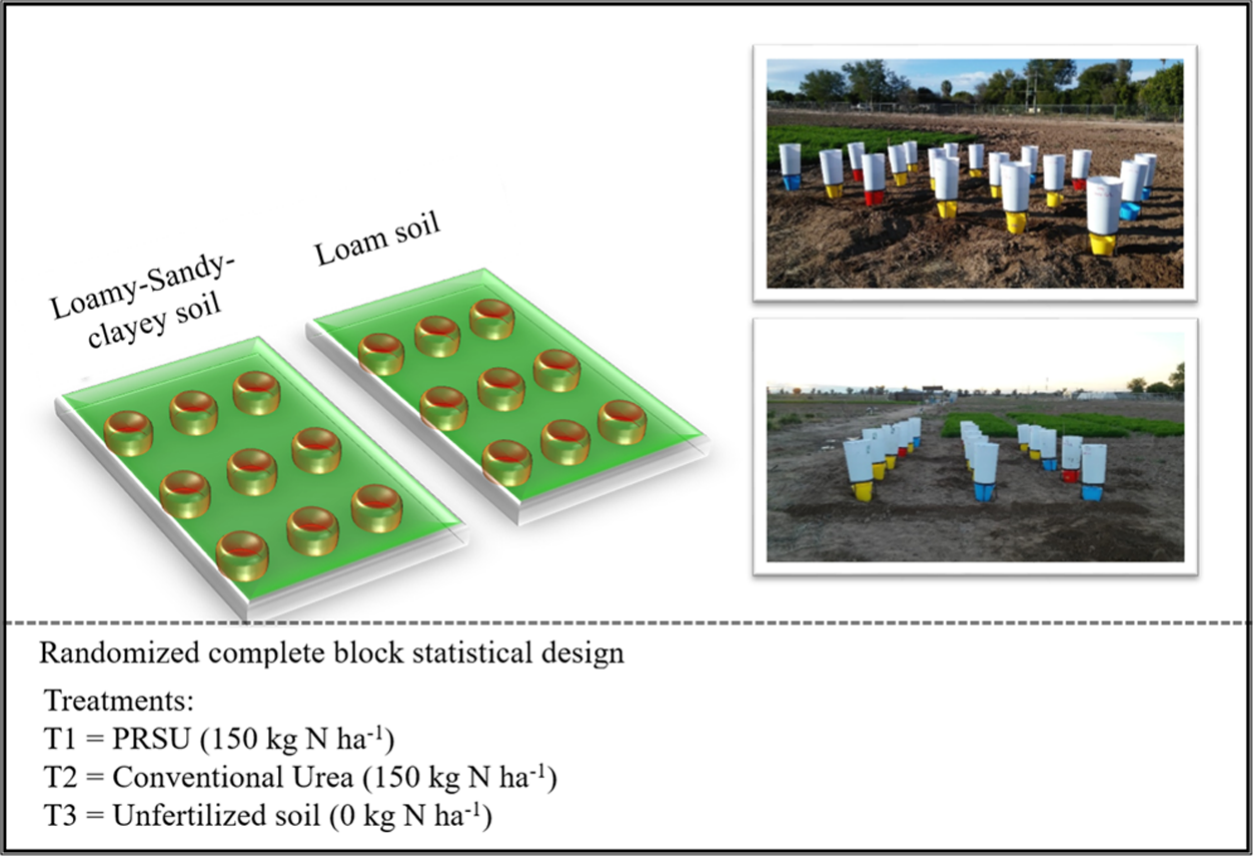
Statistical design in a randomized complete block with three treatments.

A statistical design in randomized complete block for two textures of soils (loam and loamy-sandy-clayey) with three treatments of fertilization was used (Figure 1): (1) PRS (150 kg N ha^–1^), (2) conventional urea (150 kg N ha^–1^), and (3) unfertilized soil. Each treatment was performed in triplicate. In this experiment, 18 containers for leaching were placed in the agricultural field; nine recipients contained loamy-sandy-clayey soil, and the nine remaining recipients contained agricultural loam soil. The samples were homogenized for each soil texture and placed using a soil bed depth of 30 cm, while the conventional urea and the PRSU were placed at a depth of 5 cm from the soil surface. The first irrigation was applied using an irrigation lamina of 24 cm; the second irrigation was applied after 23 days utilizing an irrigation lamina of 12 cm. After each irrigation, the leachate was collected in recipients placed under the containers for leaching for 24 h and thoroughly mixed to obtain a single sample of each treatment per block. The samples were slurred in closed plastic containers for further NO_3_^–^ and NH_4_^+^ content analysis.

**Figure 2:**
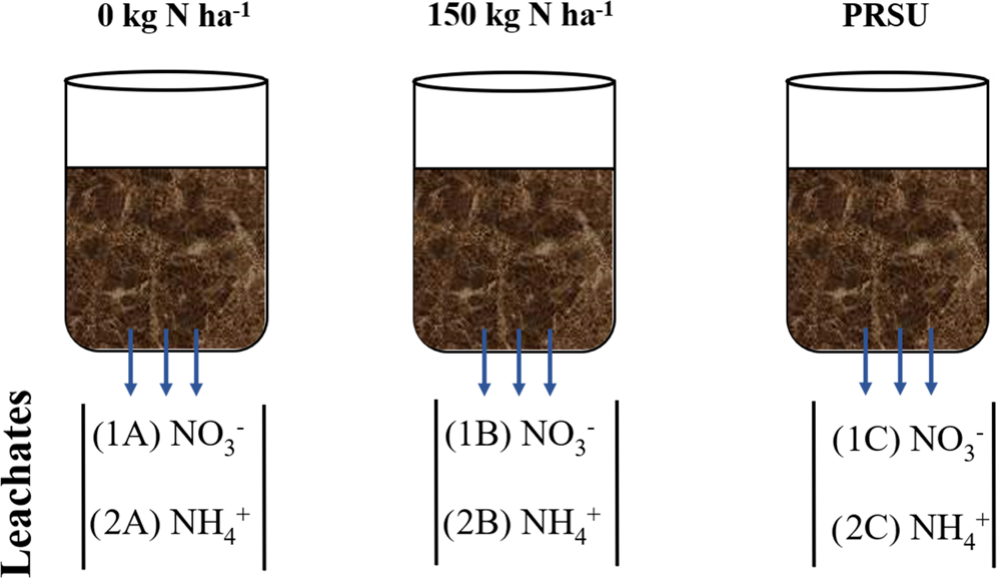
Leachates of nitrates and ammonium from the three fertilization treatments.

The nitrates (NO_3_^–^) were determined using the cadmium reduction method, and ammonium (NH_4_^+^) was determined using the Nessler method. Measurement of each analyte was carried out using a multiparameter photometer (Hanna Instruments, HI83200) and a kit (Hanna Instruments) for nitrate and ammonium determination. The environmental conditions, such as an average temperature of 20.11 °C, an average relative humidity of 55.52%, and an average precipitation of 0.43 mm, were presented during the experiment according to the Automatic Meteorological Station of Sonora. Reduction of the nitrogen losses (NO_3_^–^ and NH_4_^+^) was calculated in the leachates by subtracting the soil nitrogen losses from the samples of unfertilized soil (0 kg N ha^–1^) to the conventional urea (150 kg N ha^–1^) and PRSU (150 kg N ha^–1^) treatments (Figure 2).

The calculated values were obtained using eqs 1, 2, 3, and 4, using as reference Figure 2.
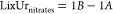
1
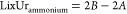
2
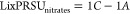
3
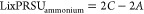
4where LixUr_nitrates_ are the nitrate values of the 150 kg N ha^–1^ treatment leachates, LixUr_ammonium_ are the ammonium values of the 150 kg N ha^–1^ treatment leachates, LixPRSU_nitrates_ are the nitrate values of the PRSU treatment leachates, and LixPRSU_ammonium_ are the ammonium values of the PRSU treatment leachates.

Also, eq 1 means the subtraction of soil nitrates, which are present as normal on the soil, to leave only the concentration of nitrates that pertains to conventional fertilization. Equation 2 means the subtraction of soil ammonium, which is present as normal on the soil, to leave only the concentration of ammonium that pertains to conventional fertilization. Equation 3 means the subtraction of the soil nitrates, which are present as normal on the soil, to leave only the concentration of nitrates that pertains to PRSU. Equation 4 means the subtraction of the soil ammonium, which is present as normal on the soil, to leave only the concentration of ammonium that pertains to PRSU. The meaning of the literals A, B, and C refers to Figure 2, where A means the leachates belonging to the kg N ha^–1^ treatment, B means the leachates belonging to the 150 kg N ha^–1^ treatment, and C means the leachates belonging to the PRSU treatment.

**Figure 3:**
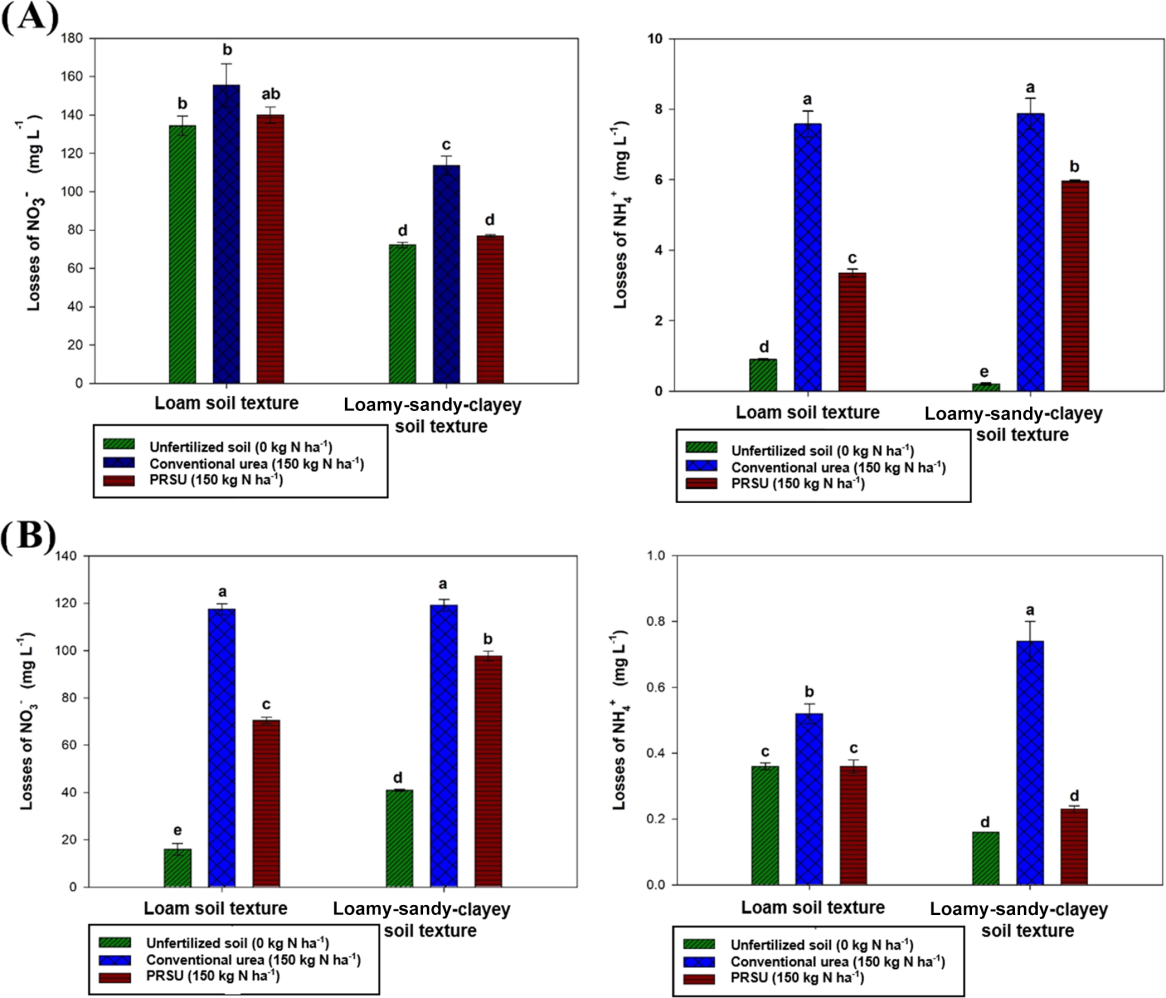
Concentrations of NO_3_^–^ and NH_4_^+^ in two types of soils during the first irrigation (a) and the second irrigation (b).

Then, the losses of the PRSU treatment were determined based on the losses exhibited in the urea treatment. With the latter, we can take the reduction of losses of nitrates and ammonium as expressed in percentages. These calculations were made using the following eqs (eqs 5 and 6)
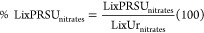
5
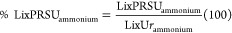
6where:

% LixPRSU_nitrates_ is the percentage of leached nitrates from the PRSU treatment.

LixPRSU_nitrates_ is the nitrate leached from PRSU treatment.

LixUr_nitrates_ is the number of nitrates leached from the 150 kg N ha^–1^ treatment.

% LixPRSU_ammonium_ is the percentage of leached ammonium from the PRSU treatment.

LixPRSU_ammonium_ is ammonium leached from the PRSU treatment (Figure 3).

LixUr_ammonium_ is the ammonium leached from the 150 kg of N ha^–1^ treatment.

### 2.4 Microbiological Analysis

Microorganism isolation was performed using the methodology reported by Vital-López et al.^23^ The soil sample to be analyzed was sieved through a mesh with a pore size of 2 mm. The soil was suspended in saline solution (0.85% w/v), and serial dilutions were carried out from 10^–1^ to 10^–6^ with saline solution (0.85% w/v). After this, Ashby’s Mannitol Agar was prepared for the growth of the microorganisms. The cultures were incubated for 4 to 5 days at 37 °C using an incubator (Thermolab model TE-I45DM). The colony-forming unit per gram of dry soil (CFU/g.d.s.) was quantified, and the observation of the macroscopic and microscopic characteristics of the colonies was conducted with Gram staining using a Binocular Microscope Olympus (model CX31RTSF) with an Infinity 1 Olympus camera (model U-CAMD3/U-TV1X-2 mounting adapter, Japan). Also, the similar colonies between both soil samples ALDUS and VE were isolated, and the similar microorganism (GMi) was used to show the effect of the PRSU on the microbial population.

#### 2.4.1 Fluorescence Microscopy

The prolonged-release system of urea (PRSU) was tested to determine its capacity to maintain microorganism viability. The PRSU was inoculated with the GMi that is present in both soil samples, and that shows similar macroscopic and microscopic characteristics. The microorganism sample that grew in Ashby’s Mannitol Agar was inoculated into nutritious broth and incubated from 24 to 48 h in an incubator (Thermolab model TE-I45DM). Afterward, 20–50 μL of nutritive broth was taken inoculated into the PRSU, the sample was added to the PRSU, and it expanded and entered into the system. The PRSU was incubated from 24 to 48 h; then, it was stained with DAPI (4′,6-diamidino-2-phenylindole). The viability of the microorganisms on the PRSU was observed using an inverted microscope (Leica Microsystems CMS GmbH Model DMi8) with a fluorescence filter (DAPI excitation 350/50 filter and emission 460/50 filter) and a cooled chamber DFC 450C (Leica). The images were processed using Overlay of fluorescence software (LAS AF version 3.1.0, Leica Microsystem).^24^

### 2.5 Statistical Analysis

Descriptive statistics (means and standard deviations) were utilized for the data obtained from the leachates. Also, for the leachates, an analysis of variance (ANOVA) was performed with a level of reliability of 95%. Tukey’s test (*p* ≤ 0.05) was used to determine significant differences between the treatments and the type of soils analyzed.

## 3 Results and Discussion

### 3.1 Agricultural Soils’ Physicochemical Properties

Loamy-sandy-clayey and loam soil texture were classified according to the textural diagram from the United States Department of Agriculture (USDA). The soil with the loamy-sandy-clayey texture exhibits a high nitrate content (NO_3_^–^), probably due to livestock activities and crop experiments in the fields. The physicochemical properties of both soils can be considered to be suitable characteristics for crop development. Table 2 shows the physicochemical properties after each fertilization treatment, so once the experimental one was concluded, it was sampled and sent to the soil analysis, that is, the second measurement after the experiment, where the loam texture does not change its texture due to an effect of the treatments of fertilization. The treatment with PRSU reveals a high content of NO_3_^–^ in comparison with that of the 150 kg N ha^–1^ treatment, with the characteristics present in both soils’ textures. In both situations, this behavior could be due to the PRSU. It is probable that during irrigation, the soil was washed, and the nitrates lost were those already contained in the soil in their natural form. However, the high content of NO_3_^–^ in the PRSU treatments reveals the prolonged release of urea behavior, i.e., the urea released was transformed through a biogeochemical process into NO_3_^–^, which is detectable after the PRSU application, the latter possibly a suitable condition due to the enrichment of the agricultural soil. In addition to the contribution of nitrogen, PRSU contributes organic matter.

**Table 2:** Physicochemical Properties of Agricultural Soils after Fertilization Treatments within a Period of 6 Months

	loam soil texture			loamy-sandy-clayey soil texture		
parameter	unfertilized soil (0 kg N ha^–1^)	conventional urea (150 kg N ha^–1^)	PRSU (150 kg N ha^–1^)	unfertilized soil (0 kg N ha^–1^)	conventional urea (150 kg N ha^–1^)	PRSU (150 kg N ha^–1^)
organic matter (%)	1.07	1.07	1.00	0.40	0.74	0.67
pH	7.45	7.17	7.31	7.30	6.84	7.17
clay (%)	25.00	23.00	23.00	19.00	19.00	19.00
silt (%)	16.00	18.00	16.00	26.00	30.00	28.00
sand (%)	59.00	59.00	61.00	55.00	51.00	53.00
NO_3_^–^ (ppm)	31.62	32.24	251.10	33.48	37.82	265.98
K (ppm)	119.00	124.00	127.00	122.00	107.00	104.00
Ca (ppm)	2180.00	2370.00	2070.00	2590.00	2500.00	2430.00
Mg (ppm)	230.00	240.00	210.00	220.00	210.00	210.00
S (ppm)	17.00	17.00	19.00	16.00	16.00	21.00
Fe (ppm)	2.30	1.50	2.20	2.20	2.00	3.80
Cu (ppm)	2.80	2.00	2.40	2.30	2.00	2.20
Zn (ppm)	0.90	0.70	0.60	0.60	0.90	1.20
Mn (ppm)	1.80	1.40	3.00	2.20	1.80	2.50
Na (ppm)	347.00	361.00	327.00	152.00	139.00	133.00

Nevertheless, the organic matter in these results does not exhibit differences, i.e., the urea treatment presented organic matter similar to the PRSU treatment. This characteristic could increase the organic matter from the PRSU if the system had been completely degraded, while the urea treatment cannot increase the organic matter because it does not possess the material necessary such as the PRSU. The PRSU would be able to maintain and increase the soil’s organic matter, mainly providing components such as C and N, regardless of the cultivation developed and the risks applied. With these characteristics, the PRSU would provide, after harvest, the necessary components (from wheat gluten proteins) to benefit the agricultural soil before sowing of any crop. During the application of long-term nitrogen in agricultural fields, acidification of soils is promoted, which affects soil degradation, fertility, and productivity. Furthermore, these changes have an effect on the microbial activity, which is affected by pH changes.^25^ However, in this study, although there is a high nitrate content after the application of PRSU, the latter does not present acidification of the soil, maintaining the pH at around 7, which is optimal for the microbial activity and the development of cereals such as wheat.

### 3.2 Nitrogen Losses

It has been reported that the oxidation of NO_2_^–^ into NO_3_^–^ by the action of *Nitrobacter* is carried out rapidly. Therefore, an accumulation of nitrites in agricultural soils is not common.^6^Figure 3a shows NO_3_^–^ and NH_4_^+^ losses during the first irrigation for the three treatments in both soil textures. Both soils present significant differences between each treatment, which can be explained as due to the particle sizes being different in the composition in every soil texture. The nitrate leachates obtained for all treatments from Loamy-sandy-clayey soil texture presented lower values in comparison with those of loam soil texture due to variation in particle size. These characteristics influence the water filtration and the drag of nitrates, i.e., the difference in particle size gives rise to the spaces between them; thus, at a certain depth, the leachate advances more quickly. The amounts of leachate for conventional urea (150 kg N ha^–1^) treatments demonstrated a high value in comparison with the PRSU (150 kg N ha^–1^) treatments. Because the urea applied in a conventional manner is in direct contact with water, its solubility properties cause quick dissolution through the soil, while the PRSU presented a polymeric barrier conferred by the wheat gluten proteins that avoided the rapid dissolution of urea into the aqueous phase. With this, the urea decreases its transport through the system to reach the outside of the PRSU, and it is not completely available for its use in the nitrogen biogeochemical process in the soil. Based on these results, the efficiency of the PRSU to reduce nitrate losses during the first irrigation was 73.75% for loam soil texture, while for ammonium, this was 63.33%. The PRSU efficiency of nitrates in loamy-sandy-clayey soil texture was 84.02%, while for ammonium, this was 24.91%. Figure 3b presents the nitrate and ammonium losses through leaching during the second irrigation for both soils. It can be observed that for unfertilized soil (0 kg N ha^–^1) treatments, there are significant differences in the nitrate and ammonium losses by the soil texture effect. The conventional urea (150 kg N ha^–1^) treatment did not present significant differences in the leaching of nitrates between both soils; however, in comparison with the first irrigation, the loamy-sandy-clayey soil texture presented an increase in the amount of nitrates. This may be due to the oxidation of nitrites into nitrates by microbial activity and also to the environmental conditions, i.e., nitrogen is an element that can be fixed from the atmosphere. Additionally, the loamy-sandy-clayey soil texture favors greater retention of nutrients and water because its structure is heterogeneous compared with the loam soil texture. The ammonium that was leached showed significant differences due to the effect of the agricultural soil texture. The PRSU treatment revealed significant differences due to soil texture, in which it can be observed that the leachate value is lower than that of the conventional urea (150 kg N ha^–1^) treatment due to the prolonged-release effect that the system presents. This effect is caused by the polymer matrix in which the urea molecules are trapped. The urea interact via hydrogen bonds with the reactive groups from the wheat gluten proteins.^13,10^ Based on the present results, it can be deduced that the effectiveness of the PRSU for nitrates during the second irrigation for the loam soil texture was 46.23%, while for the ammonium, this was 100%. The PRSU efficiency of nitrates for loamy-sandy-clayey soil texture was 27.32%, while for ammonium, this was 87.94%.

The results indicate that PRSU presented a lower amount of ammonium, and the nitrate value was higher for both soils. These results were based on the calculation of the nitrate and ammonium values obtained in eqs 1,2,3, and 4. Likewise, these partial results were used to obtain the previous percentage results of nitrate and ammonium loss decrease in each soil so that it can be discussed with the current literature regarding the decrease of nitrate and ammonium loss in leachates to the environment. In addition, the pH of the soils was maintained at close to 7 (Tables 1 and 2) for nitrification to take place, causing a lower amount of ammonium in the soil and, with this, a greater amount of nitrates. When the content of ammonium was low, the microbial activity related to nitrification was not affected. However, when there are high amounts of ammonium in the soil, there is a change in pH and microbial activity such as that of *Nitrobacter* is affected, an explanation reported in the scientific literature that helps corroborate what is found in this study.^6^

**Figure 4:**
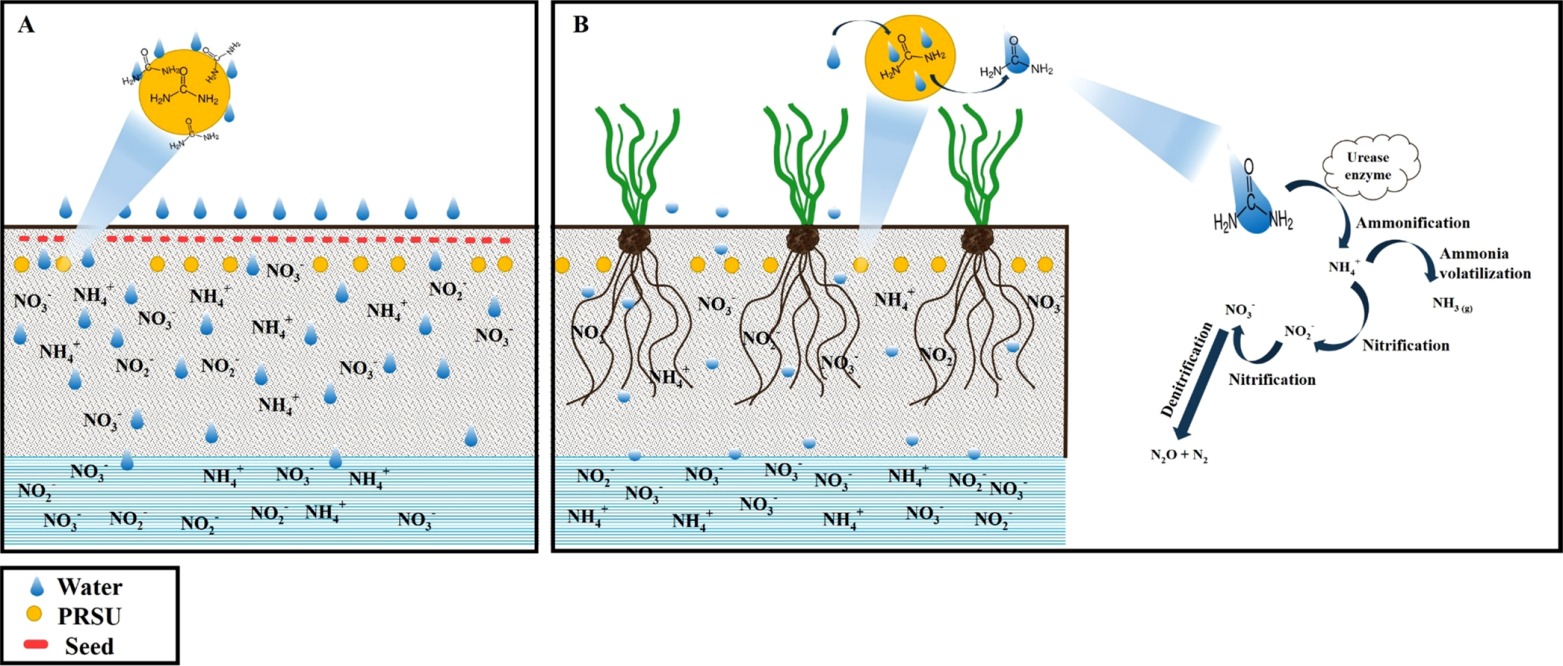
Schematic description of the PRSU: (A) first irrigation and nutrient losses and (B) second irrigation, nutrient loss, and nitrogen cycle.

Gao et al.^15^ applied a controlled-release system of urea in potato crops, where the authors obtained nitrogen use efficiency (NUE) values of 87.8–169.4 and 108.3–226.4%, using polymer controlled urea (PCU) and polymer sulfur-controlled urea (PSCU) systems. However, the authors did not specify the reduction of losses in an experiment without plants to determine the efficiency of the system in terms of reducing losses. In our study, prior to applying it to plants, we decided to use a leaching experiment to determine, without any other variable, the capacity of the system to reduce losses compared to that of the urea treatment. These results indicated that the system had the capacity to reduce N losses. According to Shibata et al.,^26^ the term nitrogen use efficiency (NUE) may be applied to agricultural practices, including the manner of fertilization. The system applied in this research may improve NUE focused on the reduction of nitrogen losses. It is important to consider that PRSU was applied only once during the experiment. This is an advantage compared with conventional fertilization practices. Considering that PRSU is a prolonged-release fertilizer, it is desirable that it provides the nutritional needs to the plant in a single application during the plant growth cycle.^7^Figure 4 presents a schematic description of N losses during the first and second irrigations; in addition, the nitrogen cycle is provided to understand the N-form transformation from the urea application in the soils. Studies where the objective is to analyze the effect of loamy and loamy-sandy-clayey textures on the performance of a PRSU against conventional urea and to measure nitrate and ammonium losses were not found in the scientific literature. It is possible to find other studies on different textures with different treatments; however, the application of a PRSU sets the tone for several investigations that could help lay the groundwork for generating scientific quality information in this section.

### 3.3 Microbiological Analysis

**Figure 5:**
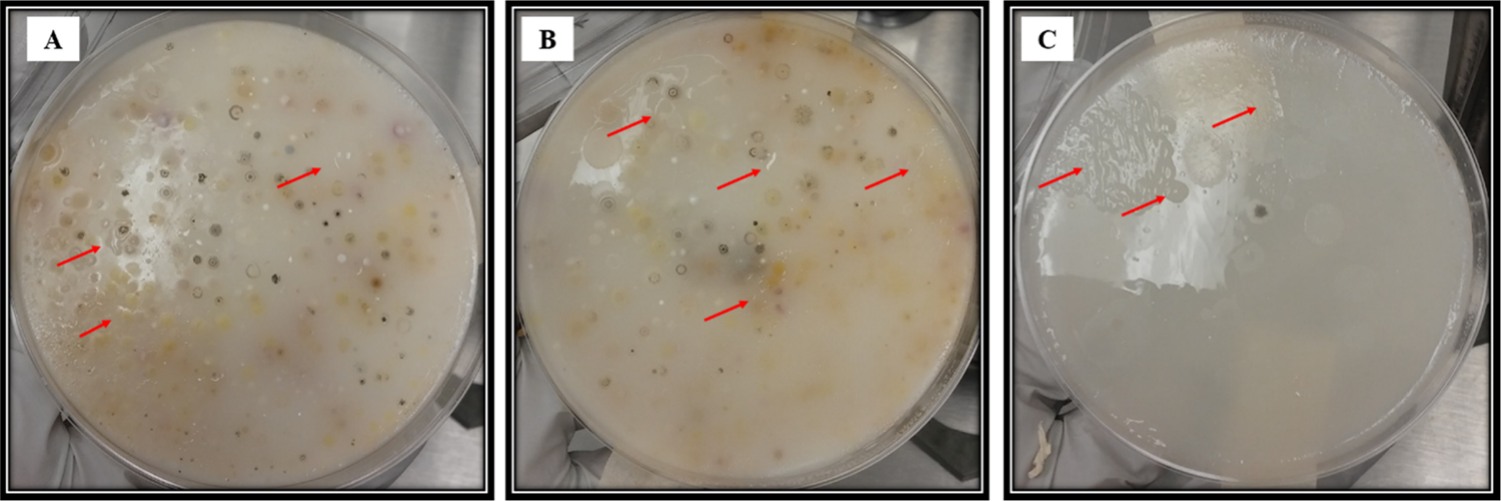
Growth of the microbial communities in Ashby’s Mannitol Agar: (A) loam soil texture, (B) loamy-sandy-clayey soil texture, and (C) similar colonies from both samples. Arrows indicate similar colonies.

Figure 5A,5B shows the microbial communities in both loam and loamy-sandy-clayey soil textures. Diverse microbial communities were observed with fungal and bacterial colonies before and after treatments. This may be due to the soils of Northwest Mexico, which are from arid lands, and the fact that these types of microorganisms are characteristic, considering that the samples were from agricultural soils. The low levels of humidity in the soil, caused by the high temperatures during most of the year, promote the growth of mainly fungi. However, the development of transparent, convex, and flat colonies in both soils can be observed. Figure 5C shows the isolated colonies described, which are similar for both soils, with the latter presenting a combination of negative and positive bacilli through Gram staining. Ashby’s Mannitol Agar is considered a specific culture medium for *Azotobacter* spp., which is classified as a plant growth promoter. This is because it promotes the nitrogen fixation, the solubilization of minerals, and phytohormone production.^27^ The growth of a strain with similar characteristics in both soils is reasonable because the soils derive from agricultural soils of the same region and are used for the development of crops.

CFU/g.d.s. for loam soil texture were 11,500 CFU/g.d.s. initially and, after the application of the system, increased to 100,000 CFU/g.d.s, which corresponds to a change of 869.56%, while loamy-sandy-clayey soil texture was initially 6,800 CFU/g.d.s. and, after the application of the system, increased to 60,000 CFU/g.d.s., which corresponds to a change of 882.35%. The variation of the microbial communities is provided by the effect of the PRSU, and it was obtained on the basis of the increase of CFU/g.d.s. These results suggest that, while PRSU is in agricultural soil, microorganisms such as *Nitrobacter* spp. and *Azotobacter* spp., among others, may be taking advantage of the organic matter, mainly wheat gluten, in order to increase its population, in turn, intervening in the nitrification process. The results discussed in Section 3.2 are consistent with the increase in the microbial population, considering that the populations of *Nitrobacter* and *Azotobacter* were increased. The former was evidenced by the high content of nitrates found after the application and the latter by its growth in a specific culture medium and its characteristics. PRSU, based on wheat gluten and urea applied in agricultural soil, can increase the microbial communities and affect the biochemical nitrogen process and form metabolites, thus aiding in microorganism growth and plant nutrition. However, these tests exhibit the effect of PRSU on microbial communities, that is, an important factor in plant growth.

#### 3.3.1 Fluorescence Microscopy

**Figure 6:**
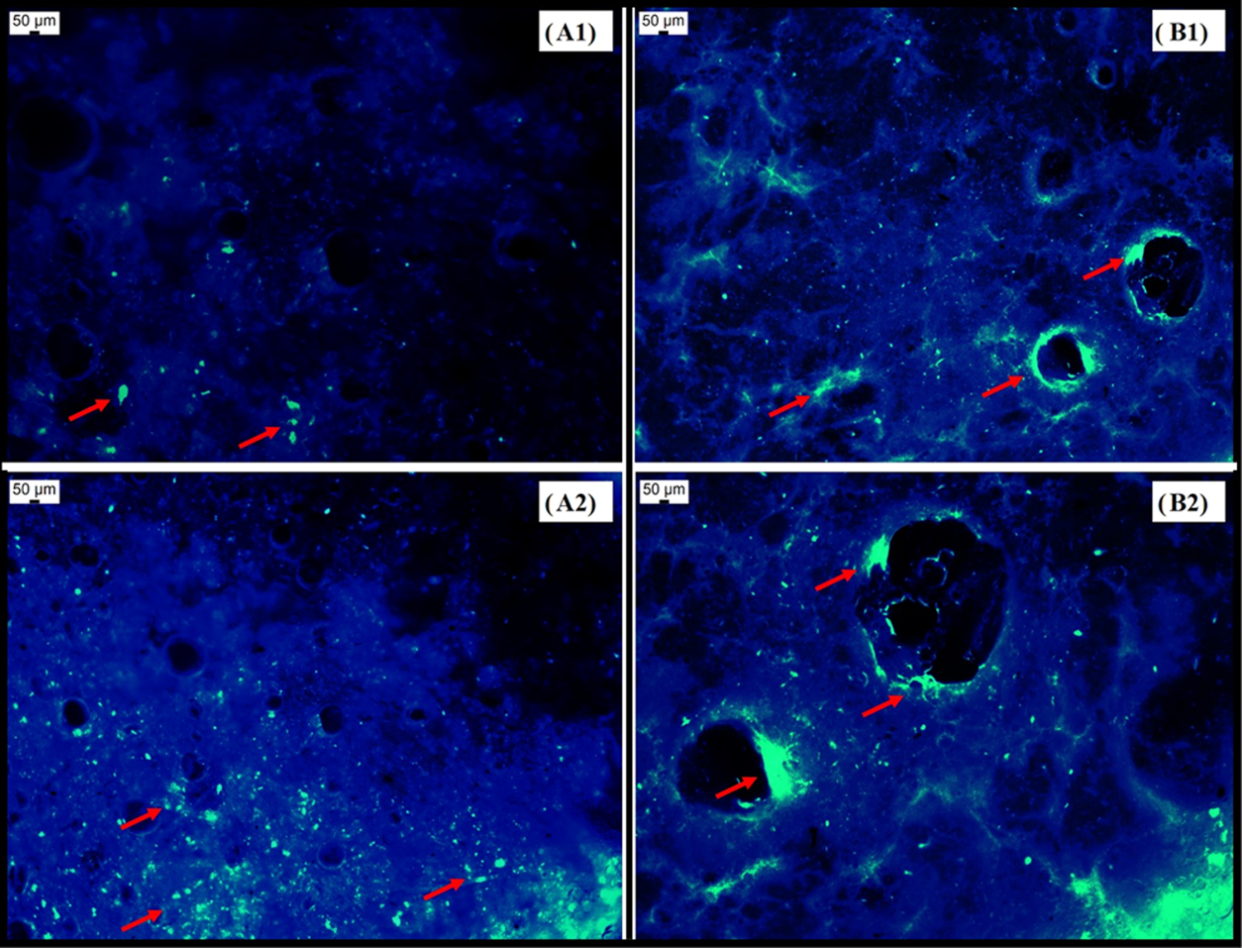
Fluorescent images using a magnification of 10× with DAPI stain: (A1) and (A2) PRS without urea, and (B1) and (B2) PRSU. Arrows indicate the viable microorganisms.

Figure 6A1,6A2 shows the fluorescent images of the PRS without urea. Clumps of microorganisms can be observed on the surface of the system. The images show a porous structure, while in Figure 6B1,6B2, we are able to observe the pores with an increased diameter, and clumps of viable microorganisms can be observed around the pores. These characteristics could be given the organic characteristics of the system due to its composition. The pores on the structure facilitate water entry into the system, and the microorganisms take advantage of the empty spaces to create suitable niches for their growth process. Also, it has been reported that several microorganisms that are plant growth promoters require growth under aerobic conditions.^28^ Additionally, the advantages of the increase of this type of microorganism lie in the production of several types of nitrogenase enzymes, which aids in nitrogen fixation, and with this, the production of phytohormones and necessary compounds for promoting plant growth.^29^

The PRSU effect on the microbial communities may benefit the interactions between the plant and the microorganisms. This effect provides suitable nutrition for the plant by interaction in the mycorrhizas, which could help with the fixation of nutrients that are not easily assimilated by the plant, also facilitating correct N nutrition from organic sources.^30^ Zeng et al.^31^ reported that nitrogen fertilization affects the bacterial diversity of the soil due to the enrichment of the soil with N, which leads to soil acidification. This causes a change in the soil pH, which hinders the growth of microorganisms. However, in this research work, we can observe the increase of nitrogen-fixing microorganisms and plant growth promoters monitored by means of a specific culture medium. At the same time, we observe that PRSU favors its growth and the amount of organic matter. Therefore, we could assume that the PRSU, according to our results, could be beneficial to agricultural soil, nitrogen fixation, and the microorganism–plant relationship.

### 3.4 PRSU Degradation under Environmental Conditions

**Figure 7:**
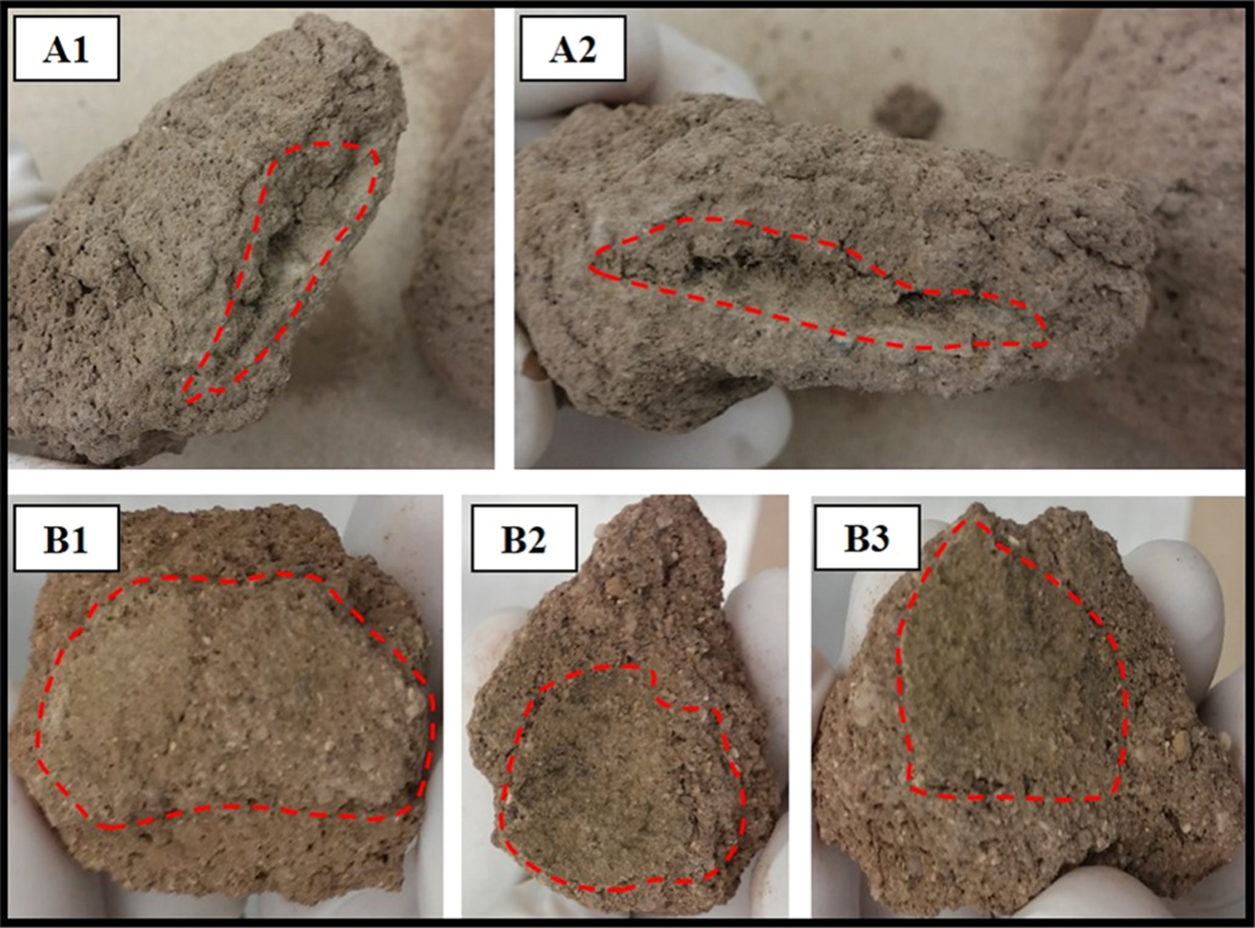
PRSU images, where the dotted red line indicates the zone of PRSU remaining after application in both soil textures: loamy-sandy-clayey (A1 and A2) and loam (B1, B2, and B3).

Figure 7 shows the PRSU after application in both soil textures (loam and loamy-sandy-clayey). Figure 7A1,7A2 presents images of the system mixed with agricultural soil, where it can be observed that part of the system was intact after the test period. Figure 7B1,7B2 shows the same effect: the system proved its capability to resist the biodegradability of the environment, i.e., it was not completely affected by the soil environmental conditions. The degradability of the material could be influenced by several factors, such as soil humidity and temperature, but mainly its biological activities.^32,33^

These results indicated to us that PRSU possesses the capacity to resist environmental conditions from the agricultural soil of the Northwest of Mexico. We could also deduce that the PRSU could provide organic matter and perhaps could maintain the release of urea until the complete degradation of the material.

## 4 Conclusions

The application of PRSU proved to produce beneficial changes in the agricultural soils, maintaining organic matter and increasing nitrate content without changes in the pH. In turn, it presented beneficial changes in the microbial communities, promoting the growth of nitrogen-fixing populations and plant growth promoters. The PRSU achieved a considerable reduction of nitrogen losses through leaching and therefore potential improvement to the environment. Also, both soil textures presented the same pattern of N loss reduction, which could be an advantage for the PRSU, which could be used in different soil textures. In summary, it is concluded that the PRSU obtained decreases the loss by leaching of nitrogen in its forms of nitrate and ammonium, regardless of the soil texture, increasing the microbial population and soil quality.

The information obtained in this investigation can aid in promoting a novel alternative to fertilization in agricultural fields using a PRSU based on wheat gluten and urea, which is able to maintain a balance in the agroecosystem of soils and, in turn, reduce the contamination caused by conventional practices of nitrogen fertilization on performing sustainable agronomic practices.
